# *Artemisia smithii* patches form fertile islands and lead to heterogeneity of soil bacteria and fungi within and around the patches in alpine meadows of the Qinghai-Tibetan Plateau

**DOI:** 10.3389/fpls.2024.1411839

**Published:** 2024-06-28

**Authors:** Hang Yang, Xiaojun Yu, Jianchao Song, Jianshuang Wu

**Affiliations:** ^1^ College of Grassland Science, Gansu Agricultural University, Lanzhou, China; ^2^ Key Laboratory of Grassland Ecosystem, Ministry of Education, Gansu Agricultural University, Lanzhou, China; ^3^ Institute of Environment and Sustainable Development in Agriculture, Chinese Academy of Agricultural Sciences, Beijing, China

**Keywords:** herbivore-avoided plant patches, *Artemisia smithii*, heterogeneous distribution, fertile islands, soil microorganism, alpine meadows

## Abstract

Herbivore-avoided plant patches are one of the initial characteristics of natural grassland degradation. These vegetation patches can intensify the spatial heterogeneity of soil nutrients within these grasslands. However, the effects of non-edible plant patches patches on the spatial heterogeneity of microorganisms have not been sufficiently studied in alpine meadows of the Qinghai-Tibetan Plateau, especially patches formed by herbaceous plants. To answer this question, soil nutrients, plant assembly, and microbial communities were measured inside, around, and outside of *Artemisia smithii* patches. These were 0 m (within the patch), 0–1 m (one meter from the edge of the patch), 1–2 m (two meters from the edge of the patch), 2–3 m (three meters from the edge of the patch), and >30 m (non-patch grassland more than thirty meters from the edge of the patch). Our results showed that *A. smithii* patches accumulated more aboveground biomass (AGB) within the patches (0 m), and formed fertile islands with the soil around the patches. Additionally, *A. smithii* patches increased soil bacterial diversity within (0 m) and around (0–1 m) the patches by primarily enriching copiotrophic bacteria (Actinobacteria), while the diversity of fungal communities increased mainly in the 0–1 m area but not within the patches. Bacterial community diversity was driven by pH, urease, nitrate nitrogen (NO_3_^−^-N), and microbial biomass carbon (MBC). The contents of soil water (SWC), soil organic matter (SOM), urease, NO_3_^−^-N, and MBC were the main factors influencing the diversity of the fungal community. This study elucidates the vegetation, nutrients, and microbial heterogeneity and their interrelationships, which are observed in fertile islands of herbivore-avoided plant patches in alpine meadows, and provides further insights into the spatial pattern of nutrients in patchy degraded grasslands.

## 1 Introduction

Grasslands are one of the dominant biomes on Earth, covering more than 40% of the land area (Petermann and Buzhdygan, 2021), and provide numerous ecosystem services and functions, such as soil and water conservation, biodiversity protection, pastoral livelihoods, and animal husbandry development (Wei et al., 2014; Qin et al., 2021). The Qinghai-Tibetan Plateau (QTP), known as the roof of the world, is a significant ecosystem dominated by natural grasslands (Sun et al., 2021). In recent years, grassland ecosystems in the QTP have experienced irrational land use and climate change, which may lead to catastrophic changes to their structure and service functions, for example, some grasslands have formed fragmented vegetation and degraded patches dominated by poisonous and herbivore-avoided species (Kéfi et al., 2014; Chen et al., 2017a), leading to a reduction in grassland productivity and the value of sustainable use. Therefore, it is important to understand the effects of patches of herbivore-avoided plants on the spatial heterogeneity of soil resources for grassland management and development (Song et al., 2020).

Plant patchiness can indicate the current state of the grassland (Heras et al., 2011; Abalori et al., 2022), and patchiness and patch self-organization have been described as processes of vegetation degradation. Lin et al. (2015) found that the expansion of degraded patches is a key feature of alpine meadows degradation. The feedback between discrete vegetation patches and soil resources has been reported to enhance both abiotic (moisture and nutrients) and biotic (microbial activity and litter decomposition) processes under vegetation, leading to the formation of resource islands or fertile islands (Ravi et al., 2007; Ding and Eldridge, 2020; Ma et al., 2024), and promoting spatial heterogeneity of soil resources. Spatial heterogeneity of soil properties plays a critical role in shaping ecosystem structure and functioning (García-Palacios et al., 2012; Hinojosa et al., 2021), especially in fertile islands formed by special plants in dryland ecosystems (Toledo et al., 2022), largely due to the mutual feedbacks between plants and soils (San Emeterio et al., 2021). Fertile islands typically have lower evaporation and temperature, and higher organic matter and microbial biomass than bare soil (Garcia et al., 2018; Li et al., 2021b). In terrestrial ecosystems, the invasion of shrubs has led to the accumulation of soil fertility under their canopy, resulting in the development of fertile islands and spatial heterogeneity of soil nutrients (Li et al., 2023). Current studies have been mainly carried out in arid or desert ecosystems characterized by low vegetation coverage and focused predominantly on shrubs. However, there is limited research on the effects of perennial herbaceous patches on soil heterogeneity, especially in alpine grassland ecosystems with continuous vegetation landscapes.

Soil sustainability is largely dependent on the composition, abundance, and activity of microorganisms (Wagg et al., 2018). Soil microorganisms are responsible for important ecosystem processes, such as participating in soil nutrient cycling, decomposing litter, and sequestering carbon (Huhe et al., 2017; Sherman et al., 2019). Several studies have been conducted on the microbial effects of fertile islands in grassland plants, with a particular focus on shrubs. The microbial biomass and diversity were higher under shrub canopy (Sun et al., 2016; Garcia et al., 2018; Li et al., 2021b), and microbial communities can influence the process of nutrient redistribution, for example, through nutrient fixation, litter decomposition, organic matter mineralization, and nutrient redistribution via fungal networks (Behie and Bidochka, 2014; Ochoa‐Hueso et al., 2017). Li et al. (2021b) found that plant patches fertile islands lead to significant heterogeneity of bacterial communities compared to soil characteristics in arid systems. These studies provide important insights into the effects of plant patches on soil microbial communities in desert and arid ecosystems. However, the information on soil microbial heterogeneity by herbaceous patches in grassland ecosystems is still scarce.

**Figure 1:**
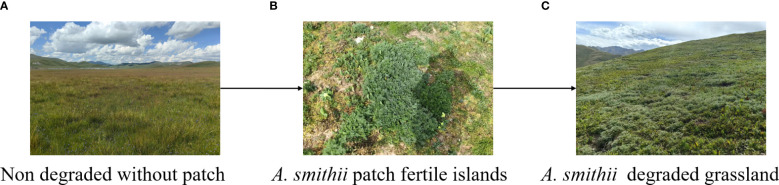
Observed phases of *Artemisia smithii* expansion in the Qinghai Tibetan Plateau.

*Artemisia smithii*, an herbaceous plant belonging to the *Artemisia* genus in the Asteraceae family. It is an unpalatable weed and is considered one of the degradation indicator species of grassland in QTP (Zhao et al., 2021). At the same time, *A. smithii* is being transformed from small-scale patches to large areas of *A. smithii* degraded grasslands (**Figure 1**). However, the research of *A. smithii* patches on grassland vegetation, soil physicochemical properties, and microorganisms has not been reported yet. Therefore, in this study, five areas within (0 meters), surrounding (approximately 1 m, 2 m, and 3 m from the edge of the patch), and non-patch grassland (more than thirty meters from the edge of the patch) of the *A. smithii* patches were selected, the plant communities and 0–10 cm soil samples were investigated and collected from each area. We hypothesized that 1) the copiotrophic microbial communities is enriched in the fertile island formed by *A. smithii* patches; and 2) the heterogeneity of soil resources radiates to the soil of patch outside, leading to an increase in soil nutrient and microbial abundance in the surrounding soil of the patch. Testing these hypotheses will help to supplement the knowledge gap of herbaceous patch fertile islands in alpine grassland ecosystems and provide strategies for grassland management in alpine grassland ecosystems.

## 2 Materials and methods

### 2.1 Study site and sampling design

The sampling site (N34°28′44″, E100°12′12″, 3756 m above sea level) located in Dawu, Maqin County, Guoluo Tibetan Autonomous Prefecture of Qinghai Province, China. The site is characterized by a typical plateau continental climate. The temperature ranges from -34.9°C to 26.6°C with an annual mean value of -0.6°C. The average annual precipitation is 443 mm. The vegetation is typical alpine meadows, dominated by *Stipa aliena*, *Elymus nutans*, and *Carex alatauensis*. The grassland is a cold-season pasture, where yaks are grazed from October to May each year, and excluded during the rest of the time.

**Figure 2:**
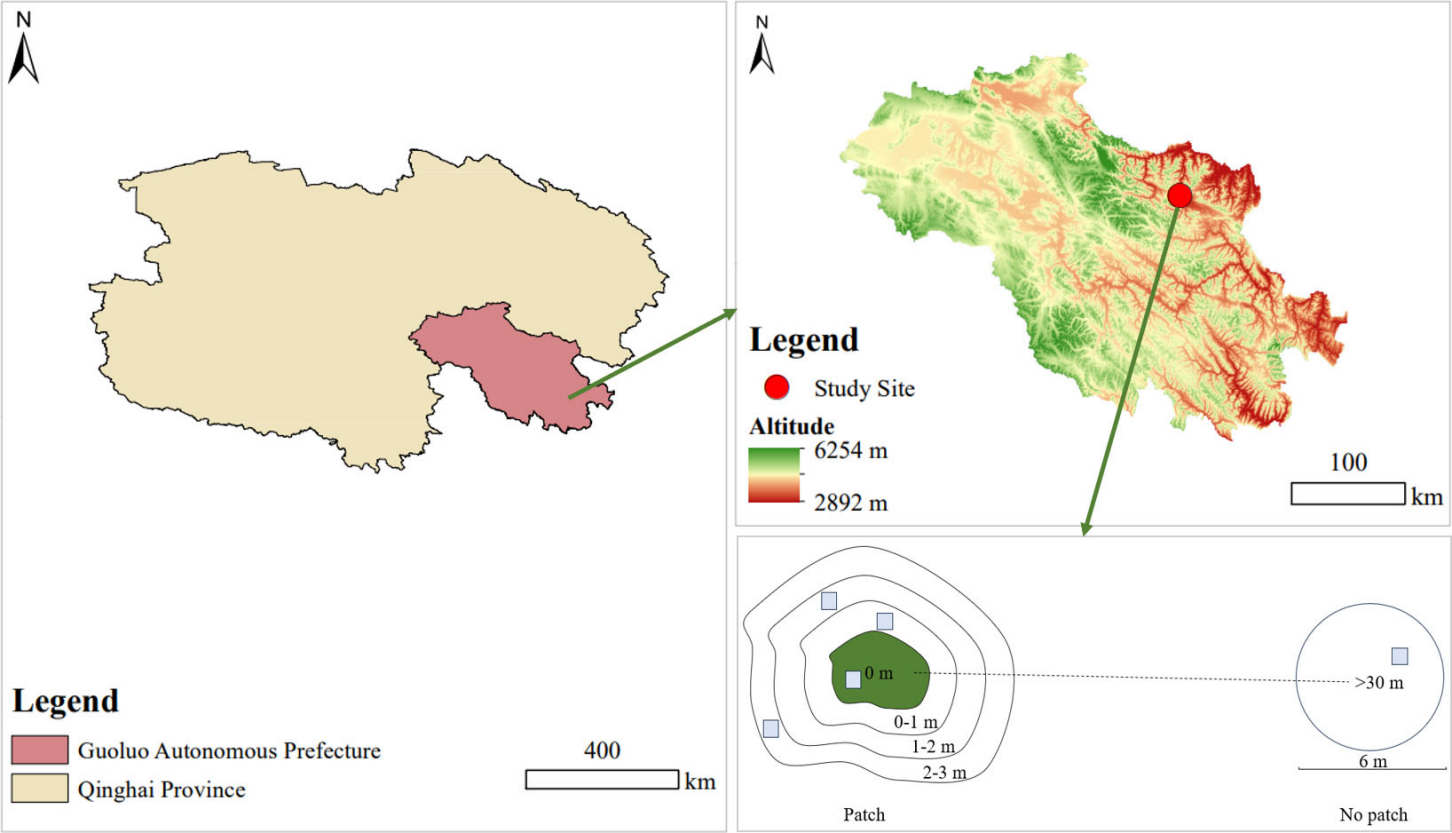
The location of the study area and the sampling sites.

In late August 2022, three patches were selected as duplicates. To eliminate errors caused by patch size and distance between patches, all three patches had an area of 8–10 m^2^ where with a coverage of over 95% of *A. smithii*, and over 30 m between each patch. Then, each patch area as the center (0 m). To examine the response of the surrounding grassland resources to the patches, the clear junction area between the patch and grassland vegetation was identified as the edge of the patch, and spreading outwards at 1 m, 2 m, and 3 m from the edge to form areas of 0–1 m, 1–2 m, and 2–3 m. Additionally, to compare the effect of the formation of *A. smithii* patches on grassland heterogeneity, three non-patch healthy grassland areas more than 30 m away from the *A. smithii* patches were selected as control and three circular areas of 8 m diameter were established, each corresponding to a patch. Five areas were designated for vegetation investigation and soil determination: 0 m, 0–1 m, 1–2 m, 2–3 m, and >30 m inside and outside the *A. smithii* patches, respectively (**Figure 2**).

### 2.2 Vegetation characteristics

Three 0.5 m × 0.5 m quadrats were randomly selected in each circular area, and species coverage and total coverage were determined using the needle-punch method in each quadrat. Ten plants of each species were selected for natural height measurements, and the mean value was the plant height in the quadrat. Species frequency was determined using the sample circle method, where sample circles were thrown 20 times in each area and the frequency of occurrence of each species was recorded. Aboveground plants within the quadrat were cut for the aboveground biomass determination, which were placed in envelopes according to each species, dried at 105°C for 30 min and then at 65°C to constant weight, and dry weight of each species was weighed, and the total biomass was the sum of each species (Ma et al., 2022). The importance value of species and plant diversity were calculated (Zhao et al., 2020; Sun et al., 2021), and the results were presented in **Supplementary Tables 1, 2**.

### 2.3 Soil sampling and properties measurements

Soil samples were collected at a depth of 10 cm using a soil auger with a 3.5 cm diameter. twenty soil cores were obtained from each area of each patch, and ten soil cores were mixed *in situ* into one composite sample to decrease errors induced by heterogeneity. Thus, ten composite soil samples were obtained in each patch (5 areas×2 composite samples). In this study, a total of 30 samples were obtained (3 patches×5 areas×2 composite samples). The soil samples were sieved through a 2 mm sieve and divided into two equal parts after removing the roots. One part was air-dried and passed through a 0.15 mm sieve to determine soil nutrients and pH. The other part soil was placed in an insulated box with ice and taken back to the laboratory for storage at -80°C for determination of soil moisture, enzyme activity, microbial biomass, and DNA extraction. Soil pH was determined in a 1:5 (soil: water) suspension with a pH-conductivity meter (Multi-Parameter PCTestr™ 35, Japan). The weighed fresh soil samples were dried at 105°C in an aluminum box until constant weight and weighed again to calculate soil water content (SWC). Soil total nitrogen (TN) was measured using the Kjeldahl nitrogen method (Bremner and Mulvaney, 1982). Soil total phosphorus (TP) was measured by colorimetrically after wet digestion with H_2_SO_4_ and HClO_4_ (Fu et al., 2004). Soil organic matter (SOM) was estimated using the potassium dichromate sulfuric acid heating method. The soil available nitrogen (AN) was estimated using the Alkali diffusion method (Sahrawat and Burford, 1982). The available phosphorus (AP) content was measured using the molybdenum blue method (Sthlberg, 1980). The nitrate nitrogen (NO_3_^−^-N) and ammonia nitrogen (NH_4_^+^-N) were determined using the NO_3_^−^-N and NH_4_^+^-N detection kit (kit number: G0319F and G0320F), the kits for determination were purchased from Suzhou Grace Biotechnology Co., Ltd., China (www.geruisi-bio.com). Urease and phosphatase activity were measured as described by Guan et al. (1991). Microbial biomass carbon (MBC), microbial biomass nitrogen (MBN), and microbial biomass phosphorus (MBP) were estimated using the chloroform-fumigation extraction method (Brookes et al., 1985; Vance et al., 1987). The results were exhibited in **Supplementary Table 3**.

### 2.4 DNA Extraction, library preparation, and illumina NovaSeq sequencing

Sequencing libraries were constructed using the NEB Next^®^Ultra™ DNA Library Prep Kit for Illumina (NEB, USA). The DNA sample was fragmented by sonication to a size of 350 bp, then DNA fragments were end-polished, A-tailed, and ligated with the full-length adaptor for Illumina sequencing with further PCR amplification. Finally, PCR products were purified (AMPure XP system), the library insert size was measured using an Agilent 2100 (Agilent Technologies Co. Ltd., USA), and the library concentration was quantified by real-time PCR.

The clustering of the index-coded samples was performed on a cBot Cluster Generation System according to the manufacturer’s instructions. After cluster generation, the library preparations were sequenced on an Illumina Novaseq 6000 platform (Illumina, San Diego, CA, USA) and paired-end reads were generated. The Raw Data of bacteria and fungi in soil samples were obtained by metagenomic sequencing using the Illumina Novaseq high-throughput sequencing platform. The clean sequences of all samples were annotated and categorized, and Bracken was used to predict the actual relative abundance of species.

### 2.5 Statistical analyses

Vegetation and soil properties were analyzed using one-way ANOVA in GraphPad Prism 9 software (GraphPad Software, San Diego, CA, USA), and statistical significance was calculated using Tukey’s HSD tests. The diversity indices (Shannon, Simpson, Chao1, and ACE) of soil fungal and bacterial communities were calculated on these rarefied sequences of tables using QIIME2 software. Nonmetric multidimensional scaling (NMDS) based on the Bray-Curtis distances and permutational multivariate analysis (PERMANOVA) were used to assess differences in soil, bacterial, and fungal community compositions across the difference distance (Che et al., 2019a). The relationships between microbial community composition (phylum and genus level) and relevant variables were calculated using redundancy analyses (RDA). To determine the relationship between plant biomass, soil physicochemical properties and soil bacterial and fungal diversity (Liao et al., 2023), Mantel test using the package of “ggcor” in R-Studio software was performed. Network construction and parameters were performed by Wekemo cloud platform (www.bioincloud.tech). The networks were visualized by the Gephi 0.10.0. All images were enhanced using Adobe Illustrator 2022.

## 3 Results

### 3.1 Vegetation and soil properties changes

The plant aboveground biomass was significantly higher in the patches than outside of patches (*p* < 0.05, **Supplementary Table 1**). The Shannon, Simpson, and evenness of the plant community inside the patches were significantly lower than outside of patches. The species important value was relatively high within the patches of *A. smithii*, while the importance values of *E. nutans*, *Poa pratensis*, *S. aliena*, and *Carex capillifolia* were higher in >30 m area (**Supplementary Table 2**). The contents of SWC, SOM, NO_3_^−^-N, and NH_4_^+^-N shown a downward trend in the 0 m, 0–1 m, 1–2 m, and 2–3 m areas, and the contents of SWC, SOM, NO_3_^−^-N, NH_4_^+^-N, and MBN was the highest in 0 m (**Supplementary Table 3**).

### 3.2 Soil microbial community composition

**Figure 3:**
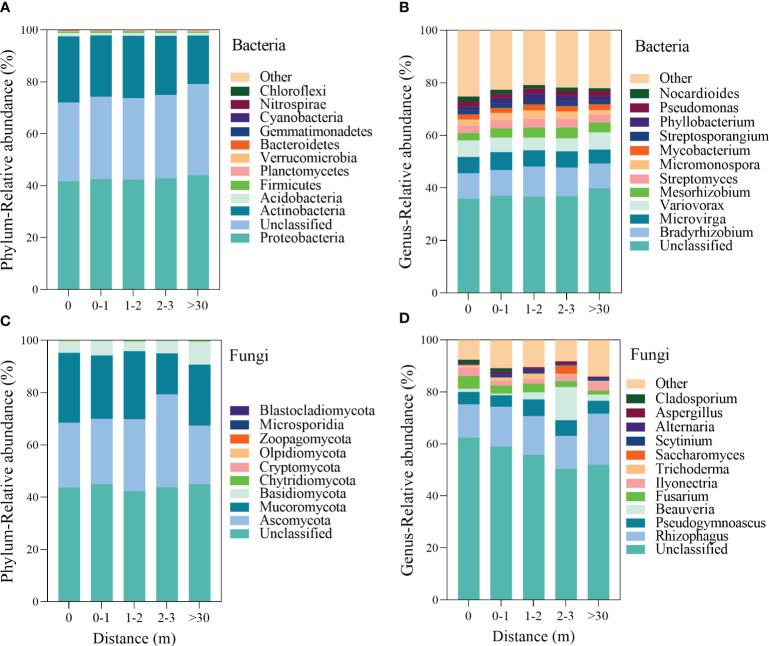
The relative abundance of the dominant bacterial **(A, B)** and fungal **(C, D)** communities at the phylum and genus level inside and outside the patches of *Artemisia smithii*.

The top 12 bacteria phyla were Proteobacteria, Actinobacteria, Acidobacteria, Firmicutes, Planctomycetes, Verrucomicrobia, Bacteroidetes, Gemmatimonadetes, Cyanobacteria, Nitrospirae, and Chloroflexi (**Figure 3A**), the Actinobacteria was higher in 0 m area (*p*<0.001, **Supplementary Figure 1B**). At the genera level of the bacterial community, the abundance of *Bradyrhizobium*, *Microvirga*, *Variovorax*, *Mesorhizobium*, *Streptomyces*, *Micromonospora*, *Mycobacterium*, *Streptosporangium*, *Phyllobacterium*, *Pseudomonas*, and *Nocardioides* was relatively higher (**Figure 3B**). The relative abundance of *Bradyrhizobium*, *Mesorhizobium*, and *Micromonospora* were a unimodal increasing trend with the increased distance from the patch (**Supplementary Figure 2**).

The dominant soil fungal communities at the phylum level were Ascomycota, Mucoromycota, Basidiomycota, Chytridiomycota, Cryptomycota, Olpidiomycota, Zoopagomycota, Microsporidia, and Blastocladiomycota (**Figure 3C**). The relative abundance of Ascomycota exhibited a unimodal increasing trend, reaching a peak in 2–3 m area. (**Supplementary Figure 3**). At the genus level of the fungi, the abundance of *Rhizophagus*, *Pseudogymnoascus*, *Beauveria*, *Fusarium*, *Ilyonectria*, *Trichoderma*, *Saccharomyces*, *Scytinium*, *Alternaria*, *Aspergillus*, and *Cladosporium* was higher (**Figure 3D**). The relative abundance of *Fusarium* exhibited a decreasing trend with increasing distance from the patch (**Supplementary Figure 4**).

### 3.3 Soil microbial community α and β-diversity

**Figure 4:**
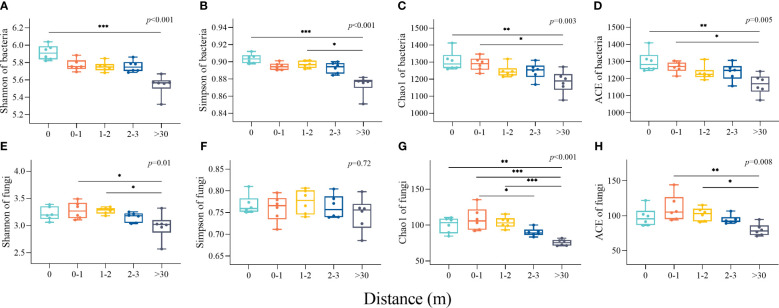
The characteristics of soil bacterial and fungal diversity and richness indices inside and outside the patches of *Artemisia smithii*. **(A–D)** were Shannon, Simpson, Chao1, and ACE indexes of bacterial communities, respectively; **(E–H)** were Shannon, Simpson, Chao1, and ACE indexes of fungal communities, respectively. (*, p<0.05; **, p<0.01; ***, p<0.001).

The diversity indexes (Shannon and Simpson) and richness indexes (Chao1 and ACE) of bacterial communities were both the highest in 0 m area and the lowest in >30 m area. The bacterial α-diversity indices in the 0 m area were significantly greater than the >30 m (**Figures 4A, B**). The Shannon, Simpson, Chao1, and Ace indices of soil fungal communities were a unimodal increasing trend with increasing distance from the patches. The Shannon indexes in the 0-1 m and 1-2 m areas were significantly higher than the >30 m (*p*<0.05, **Figure 4E**), and the Chao1 indexes in the 0 m, 0–1 m, and 1–2 m areas were significantly higher than the >30 m (**Figure 4G**).

**Figure 5:**
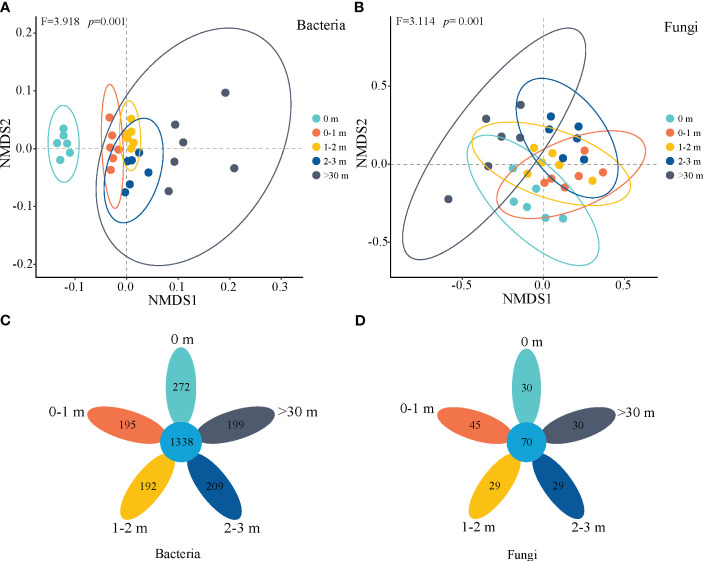
Non-metric multidimensional scaling (NMDS) of bacterial **(A)** and fungal **(B)** communities based on the Bray-Curtis distances and flower plot of bacteria **(C)** and fungi **(D)** inside and outside the patches of *Artemisia smithii*.

Based on the NMDS analysis of Bray Curtis distance, the β-diversity of soil bacterial and fungal communities strongly responded to inside and outside the patch (*p*=0.001, **Figures 5A, B**), and the bacterial community in 0 m area exhibited significant heterogeneity compared to other treatments (**Figure 5A**). A total of 1338 common soil bacterial species were identified, with 272, 195, 192, 209, and 199 endemic species observed at depths of 0 m, 0–1 m, 1–2 m, 2–3 m, and >30 m, respectively. The number of fungal common species was 70, while the number of endemic species was 30, 45, 29, 29, and 30, respectively.

**Figure 6:**
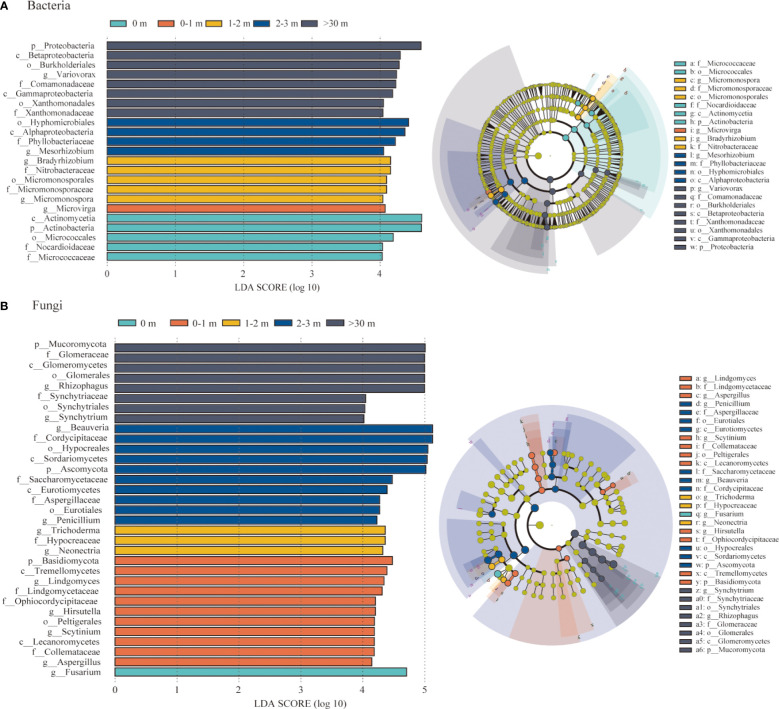
Results of linear discriminant analysis (LDA) effect size (LEfSe). Bacterial **(A)** and fungal **(B)** taxon nodes that significantly enriched in each treatment were shown in color, and branch areas were shaded according to the highest ranked variety for that taxon. Only taxa with LDA score > 4.0 were shown.

To identify the bacterial and fungal taxa responsible for community differentiation, biomarkers of each treatment were identified by LEfSe analyses for the five areas soil (**Figure 6**). A total of 23 bacterial and 33 fungal clades exhibited statistically significant differences among the different treatments. In the 0 m area, there was a significant increase in the abundance of Actinobacteria and Actinomycetia. Only *Microvirga* exhibited a significant enrichment in 0–1 m area, while the Micromonosporales, Bradyrhizobium, and Nitrobacteraceae exhibited substantial enrichment under the 1–2 m area. In the >30 m area, *Variovorax*, Comamonadaceae, Burkholderiales, Betaproteobacteria, Xanthomonadaceae, Xanthomonadales, Gammaproteobacteria, and Proteobacteria were significantly enriched (**Figure 6A**). Regarding the soil fungal communities, *Fusarium* was identified as a biomarker in 0 m area. Furthermore, Lecanoromycetes, Lindgomycetaceae, *Aspergillus*, Ophiocordycipitaceae, Tremellomycetes, and Basidiomycota were significantly enriched in 0–1 m. The *Synchytrium*, Synchytriaceae, Synchytriales, *Rhizophagus*, Glomeraceae, Glomerales, Glomeromycetes and Mucoromycota were enriched in >30 m area (**Figure 6B**).

### 3.4 Relationships between microbial variables and environmental factors

**Figure 7:**
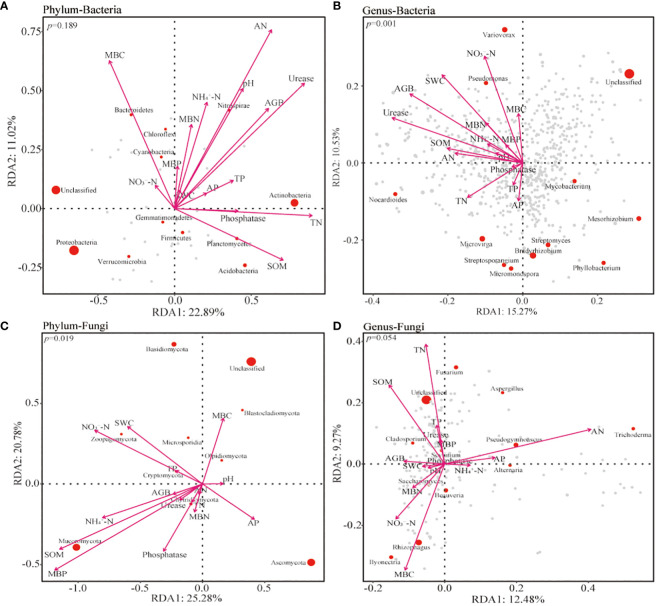
The redundancy analysis (RDA) of the bacterial **(A, B)** and fungal **(C, D)** communities at the phylum and genus level with environmental factors inside and outside the patches of *Artemisia smithii*. SWC, soil water content; TN, total nitrogen; TP, total phosphorus; SOM, soil organic matter; AN, alkali-hydrolyzable nitrogen; AP, available phosphorus; NO_3_^−^-N, nitrate nitrogen; NH_4_^+^-N, Ammonia nitrogen; MBC, microbial biomass carbon; MBN, microbial biomass nitrogen; MBP, microbial biomass phosphorus.

**Figure 8:**
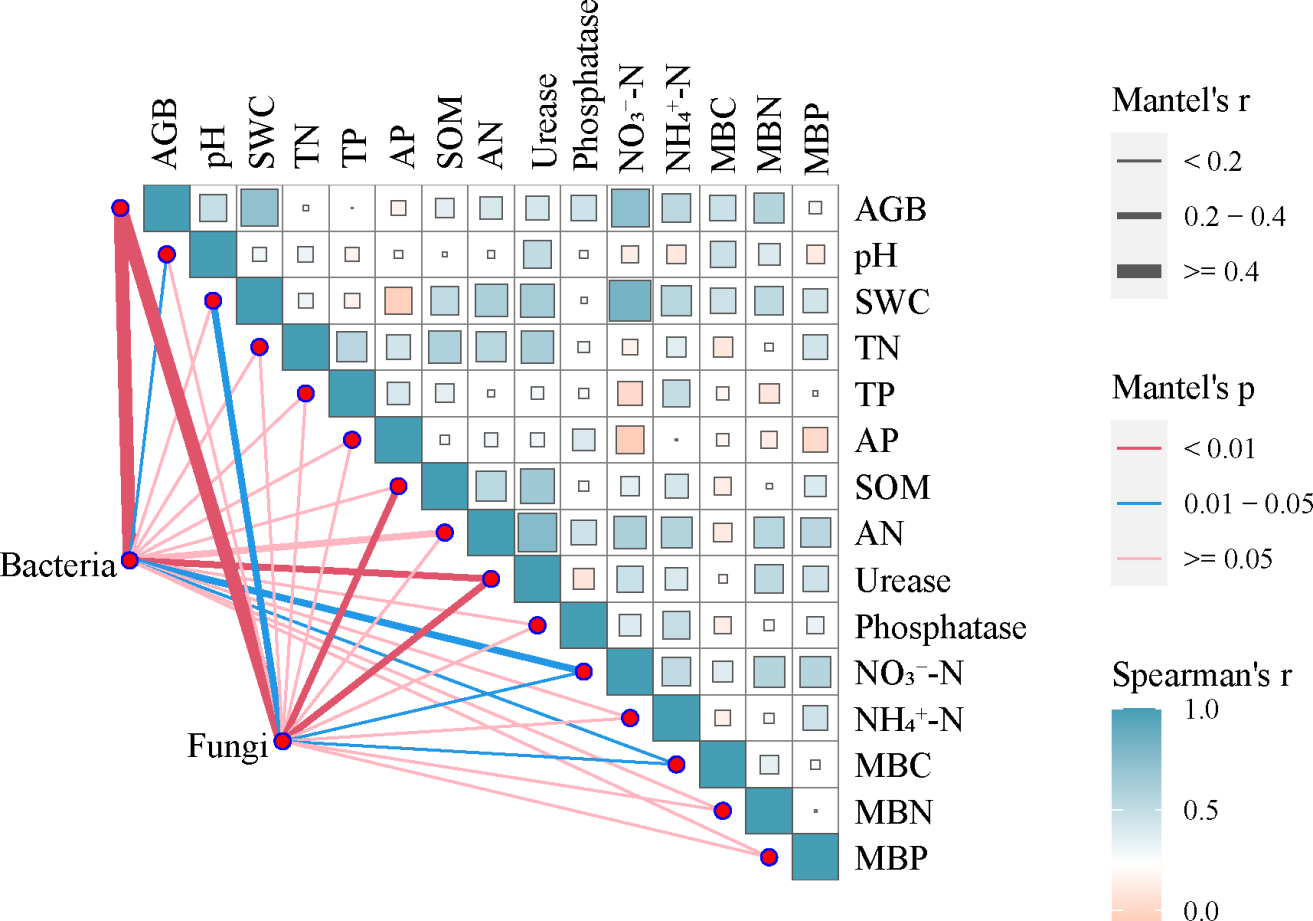
Mantel test for the correlation between community composition and environmental variables for bacteria and fungi based on α-diversity. Pairwise comparisons of environmental factors with a color gradient denoting Spearmen’s correlation coefficient. Edge width corresponds to the Mantel’s r statistic for the corresponding distance correlations, and edge color denotes the statistical significance. The solid and dotted lines indicate positive and negative effects between the variables, respectively. SWC, soil water content; TN, total nitrogen; TP, total phosphorus; SOM, soil organic matter; AN, alkali-hydrolyzable nitrogen; AP, available phosphorus; NO_3_^−^-N, nitrate nitrogen; NH_4_^+^-N, Ammonia nitrogen; MBC, microbial biomass carbon; MBN, microbial biomass nitrogen; MBP, microbial biomass phosphorus.

The relationship between soil microorganisms and environmental variables at different treatments was analyzed using RDA analysis. The results indicated that AGB, pH, TN, AN, urease, SOM, and MBC had significant effects on the community composition of soil bacteria at the phylum level. At the genus level, AGB, SWC, TN, AN, AP, SOM, urease, NO_3_^−^-N, NH_4_^+^-N, MNC, and MBN had a significant effect on bacterial community (**Figures 7A, B**). These variables were more concentrated towards the 0 m soil bacterial community (**Supplementary Figures 5A, B**). At the phylum level, the composition of fungal communities was significantly influenced by SOM, MBP, and NO_3_^−^-N (**Figure 7C**). The TN, AN, SOM, and MBC had a significant impact at the genus level of fungal communities (**Figure 7D**). The α-diversity matrix of bacterial communities was significantly affected by AGB, pH, urease, NO_3_^−^-N, and MBC. Similarly, AGB, SWC, SOM, urease, NO_3_^−^-N, and MBC significantly affected fungal diversity using the Mental test (**Figure 8**).

### 3.5 Soil microbial-related networks

**Figure 9:**
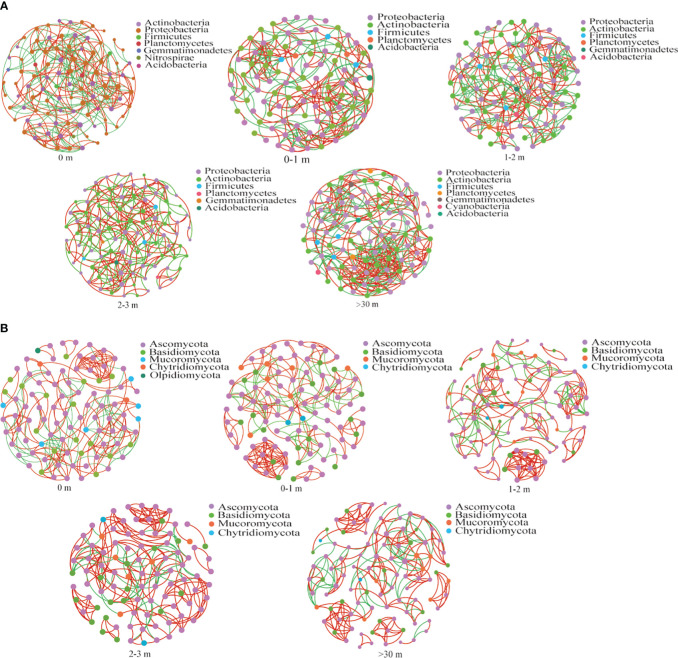
The dynamic changes in soil microbial networks. The co-occurrence network was investigated under different treatments for soil bacterial community **(A)**, fungal community **(B)**. The top 100 microorganisms with relative abundance ranking at the genus level were selected for correlation analysis (Spearman correlation coefficient p>0.5, p<0.05). Each node in the network has a different color based on its phylum, and node size was weighted according to the degree. Red links represent positive correlations between nodes, and green links represent significant correlations between nodes.

The relationship between soil bacterial communities was dominated by Proteobacteria, Actinobacteria, Firmicutes, Planctomycotes, and Acidobacteria (**Figure 9A**), and the bacterial community in >30 m exhibited higher average degree and edges (**Figure 9**; **Supplementary Table 4**). In comparison to the response of bacteria to *A. smithii* patches, the correlation of the fungal community was found to be weaker (**Figure 9B**).

## 4 Discussion

### 4.1 *Artemisia smithii* patches form soil fertile islands

The *A. smithii* patches created fertile island and induced spatial heterogeneity in soil properties. Our study showed that more water and nutrients were accumulated within *A. smithii* patches (**Supplementary Table 3**). On the one hand, water evaporation due to canopy shading was reduced by the high plant density within the patch, which resulted in more water accumulation in the patches, and on the other hand, the patches induced a redistribution of water resources, which resulted in more water moving towards the patches soils (Yan et al., 2019). In addition, the soil water content was significantly higher in the >30 m area than in the 1–2 m and 2–3 m areas, probably because the plant aboveground biomass was higher in the >30 m area than in the 0–1 m, 1–2 m, and 2–3 m areas (**Supplementary Table 1**), and less water was evaporated. Cao et al. (2016) showed that there was higher soil water content under the taproot of *Haloxylon ammodendron*, and the soil water content decreased with distance, which is consistent with our study.

In arid or semi-arid ecosystems, fertile islands or resource islands are formed under the shrub canopy (Li et al., 2021b). In our study, the patch of herbaceous plant *A. smithii* also caused fertile islands in alpine meadows ecosystems, with higher TN, SOM, AN, NO_3_^−^-N, and NH_4_^+^-N contents clustered under patches. Usually, fertile islands are due to nutrient aggregation caused by litter decomposition, and higher root secretions under the plant canopy (Diedhiou-Sall et al., 2013; Cao et al., 2016). In alpine meadows, *A. smithii* is not browsed by domestic animals, so the accumulation of litter in patches may result in the accumulation of water and nutrients that create fertile islands.

### 4.2 *Artemisia smithii* patches lead to an increase in microbial abundance within and around the patches

Fertile islands by shrub canopy alter soil microbial community structure through plant litter, root exudates, and shading from solar radiation (Loreau et al., 2006; Berg et al., 2015), previous studies have found that plant fertile islands affect soil bacterial communities, with an increase in the abundance of bacterial taxa with specific trophic strategies and functional potentials, and the diversity and abundance of bacterial communities in fertile islands formed by taproot and canopy plants are higher than bare land or intercanopy spaces (Xiang et al., 2018; Li et al., 2021b; Toledo et al., 2022). In this study, the relative abundance of Proteobacteria was higher in >30 m, and the relative abundance of Actinobacteria decreased with increasing distance from the patch (**Figure 3A**; **Supplementary Figure 1**). However, Proteobacteria and Actinobacteria showed different trends as copiotrophic bacteria. Dai et al. (2018) found that long-term nitrogen fertilization increased the relative abundance of Proteobacteria and Actinobacteria, suggesting that nutrient accumulation caused beneficial for Actinobacteria colonization, while >30 m had higher Proteobacteria abundance than inside and around the patches (**Supplementary Figure 1**), which may be due to external environmental disturbance (reduced vegetation and soil nutrients), and copiotrophic bacteria Proteobacteria face habitat changes and adopt an R-type strategy for large-scale reproduction (Li et al., 2021a). These results are not entirely consistent with our first hypothesis, with only Actinobacteria was enriched under *A. smithii* patches.

Microbial community diversity is altered by plant patches, e.g., positive feedback, negative feedback, and no effect. Li et al. (2021b) showed that soil bacterial community diversity was higher in the center of fertile islands of *Artemisia* and *Hedysarum* patch than edges and periphery. Doniger et al. (2020) found that soil bacterial communities in bare land without vegetation had higher diversity than in the soil below vegetation. In addition, studies have shown that there is no significant difference in the diversity of soil bacterial communities under shrub patches and in the soil between patches (Sun et al., 2016). In this study, genus-level bacterial communities were also found to respond to *A. smithii* patch fertile islands, suggesting that plant-soil feedback processes can cause changes in bacterial community composition after patch formation. The α-diversity of bacterial communities (Shannon, Simpson, Chao1, and ACE indices) in the >30 m area was lower (**Figure 4**). This indicates that the *A. smithii* patches significantly lead to soil bacterial community heterogeneity in alpine meadows ecosystems.

Fertile islands by plant patches are not only a result of plant activity (Reynolds et al., 1999), but also a comprehensive effect of microbial community activity under the plant canopy (Cleveland et al., 2013). Plant traits indirectly regulate the fertile islands patches through microbial communities (Ochoa‐Hueso et al., 2017). The *A. smithii* patches in this study affected the soil fungal community composition, with Ascomycota relative abundance higher and Mucoromycota lower in the 2-3 m, and inconsistent trends for other phylum-level fungi, suggesting that microenvironmental alterations caused by the *A. smithii* patches lead to either positive or negative associations between fungal communities, and resulting in either co-operation or competition. *A. smithii* patches altered the diversity of fungal communities in the soil immediately surrounding the patches, with fungal Shannon, Chao1 and Ace indices higher in the 0–1 m and 1–2 m than in the >30 m (**Figure 4**), this suggests, on the one hand, nutrient-rich soils are not conducive to fungal colonization, and previous studies have found a significant increase in fungal α-diversity in lower nutrient bare land patches (Che et al., 2019a). On the other hand, these results indicate that bacterial and fungal community diversity responded differently to the *A. smithii* patches, with the formation of *A. smithii* patches leading to an increase in bacterial diversity within the patches, whereas an increase in fungal diversity was observed in the soil surrounding the patches. In addition, in this study, the β-diversity of the 0 m bacterial community was relatively separated from the other treatments, while the β-diversity of the fungal community was clustered together (**Figure 5**), which further suggesting that *A. smithii* patches caused spatial heterogeneity of soil microorganisms, which may be related to the heterogeneity of the vegetation and the nutrients.

### 4.3 *Artemisia smithii* patches lead to the heterogeneity of soil microbial communities closely relate with plant and soil nutrition

*A. smithii* patches resulted in the heterogeneous distribution of most physicochemical properties and significant differences in biological properties, such as urease, MBC, MBN, and MBP. Iwaoka et al. (2017) found that fertile islands accumulated more soil TN, and San Emeterio et al. (2021) showed that *B. rupestre* patches had higher urease, MBC, and MBN under the patches than the areas without patches. Plant diversity promote the diversity of soil microbes by increasing the diversity of food resources (Prober et al., 2015). *A. smithii* patches affected plant structure and diversity within and around the patch, with aggregation of *A. smithii* resulting in higher biomass and lower Shannon and Simpson indices than outside the patches (**Supplementary Table 1**). The important values of most grasses were higher in the >30 m, while poisonous plants were higher around the *A. smithii* patch (**Supplementary Table 2**). This suggests that the plant-soil mutual feedback process caused by fertile islands may alter species richness and diversity, while domestic grazing in alpine meadows may trigger the invasive behaviors of some perennial weeds (Vinton and Goergen, 2006; van der Putten et al., 2013), and changes in plant diversity may affect soil microbial structure and diversity.

The enrichment or reduction of soil physicochemical content and plant biomass were significantly correlated with microbial community structure and diversity. RDA analyses showed that changes in bacterial community composition at the phylum-level were largely attributed to AN, MBC, urease, TN, and SOM, and that genus-level bacterial community composition was significantly influenced by environmental factors, with urease, AGB, SWC, and NO_3_^−^-N being the main factors and having a positive feedback effect in 0 m. Actinobacteria and Bacteroidetes have positively correlated responses to TN and MBC, respectively, and Actinobacteria and Bacteroidetes have been reported to be copiotrophic bacteria (Li et al., 2021b). In the present study, the relative abundance of Actinobacteria was higher in the 0 m and lower in the >30 m. This suggests that *A. smithii* patches created nutrient-rich environment that will attract some of the copiotrophic bacteria into the patch, while more plant litter and root exudates within the patch will provide a better environment for the copiotrophic microorganisms to colonies in the patches (Schlesinger and Pilmanis, 1998). Soil phylum-level fungal communities were influenced by MBP, SOM, NO_3_^−^-N and NH_4_^+^-N, and genus-level fungal communities were more influenced by AN, TN, MBC, and SOM. Due to *A. smithii* patches have higher biomass and are not browsed by livestock, more litter remains in the patches for more time and may be incorporated into a stable organic matter pool. This process is mediated by more active and abundant fungal communities (Ochoa‐Hueso et al., 2017). In this study, Mucoromucota and Cladosporium were significantly positively correlated with SOM and were higher in the 0 m area, while Ascomycota and most nutrients showed a negative response. Ascomycota is one of the oligotrophic groups and can survive and adapt in resource or nutrient deficient environments (Chen et al., 2017b). The relative abundance of Ascomycota was a unimodal increasing trend with distance outward, which is consistent with the trend of most nutrients. In addition, previous studies have shown that fungi usually dominate in oligotrophic habits and arid conditions where carbon or nutrient resources are scarce (Clemmensen et al., 2014), but in our study fungal community diversity did not show a completely opposite trend to soil sample nutrients, suggesting that grassland type may influence microbial community structure. Yao et al. (2023) showed that microbial diversity was regulated by grassland type, and that fungal diversity tended to be higher in typical grasslands and desert grasslands. Ma et al. (2023) demonstrated that soil fungal diversity was higher in warm and temperate ecotone than in warm-temperate. These results suggest that changes in vegetation and soil sample heterogeneity due to grassland type are the factors that dominate microorganism changes.

According to Mantel analysis (**Figure 8**), heterogeneity in bacterial and fungal α-diversity were induced by *A. smithii* patches was correlated with vegetation and soil physicochemical and biological properties. The aboveground biomass was found to significantly affect bacterial and fungal α-diversity (rM≥0.4, pM<0.01), while the bacterial community diversity was driven by pH, urease, NO_3_^−^-N, and MBC. The contents of SWC, SOM, urease, NO_3_^−^-N, and MBC were the main factors influencing the diversity of the fungal community. In this study, the α-diversity of fungal community showed a unimodal increasing trend with distance from the patch outwards. In contrast, NO_3_^−^-N and NH_4_^+^-N demonstrated an opposite trend to the fungal α-diversity, suggesting that the aggregation of water under *A. smithii* patches may reduce the oxygen in the soil, thereby limiting fungal development.

### 4.4 Co-occurrence of soil bacteria and fungi Networks respond to *Artemisia smithii* patches

Microbial network analysis helps to analyze the interrelationships between communities in a shared ecological niche (Schmid et al., 2017). In this study, the bacterial community had higher edges and connectivity degree in >30 m area (**Figure 8**; **Supplementary Table 4**), while the microbial network relationships were similar between around and within the patch, suggesting that microorganisms enhanced co-operation to improve survival in lower nutrient environments, and that the *A. smithii* patches made the characteristics of the surrounding soil organisms similar to those in the patch, which might create a microenvironment favored by *A. smithii*. Du et al. (2022) study showed that the formation of complex networks usually requires a nutrient-rich environment, which is inconsistent with the present study, and the possible explanation may be different bacterial species respond to nutrient-rich environments with different strategies, whereas nutrient-rich environments may allow bacterial communities to maintain high abundance and diversity without requiring more information exchange. In addition, fungal microbial network relationships did not differ significantly, which may have more fungal species in the dormant state. The dormant fungi usually have fewer associations with other soil microorganisms, and although the diversity and abundance of fungal communities in the soil were altered, an increase in the number of dormant fungal species will inevitably reduce the complexity of fungal interactions (Deng et al., 2012; Che et al., 2019a, 2019b).

### 4.5 The soil biotic and abiotic heterogeneity caused by *Artemisia smithii* patches may be beneficial for patch expansion

Consistent with our second hypothesis, the heterogeneity of soil resources caused by *A. smithii* patches radiated to the surrounding soil of patches, with an increase in nutrient, bacterial, and fungal α-diversity in the 0–1 m, 1–2 m areas compared to >30 m. In alpine meadow ecosystems, the fertile island of *A. smithii* patch may be an important mechanism for ecosystem self-organization or resistance (Yan et al., 2019), the *A. smithii* patch may create a stable mini-ecosystem under grazing conditions. Previous studies have shown that fertile island is highly self-reinforcing and can expand patch area by absorbing more resources (Eldridge et al., 2011; Yan et al., 2019). The fertile accumulation caused by *A. smithii* patches may further consolidate its own dominance in the patches, and some studies have shown that fast-growing plant species become dominant in the community after fertilization, while the abundance of slow-growing plants will be reduced (Chen et al., 2011; Holub et al., 2012). This suggests that *A. smithii* patch may strengthen the stability and competitiveness of communities under human activity disturbance, and it is possible that the soil nutrient, biological, and microbial heterogeneity caused by *A. smithii* patch in the patches surrounding may create more favorable conditions to expand patch area. In addition, allelopathy is an important mediator among plant species in managed ecosystems, plants produce and release allelochemicals that interfere with the establishment and growth of interspecific plants and mediate below-ground ecological interactions and plant-soil feedbacks (Xu et al., 2023). The originally dominant species are gradually being replaced by other plants with allelopathic traits and display aggression towards surrounding plants, Li et al. (2011) found that *A. frigida* inhibited seed germination and seedling growth by releasing volatile allelochemicals. Therefore, the driving factors for the formation and expansion of *A. smithii* patch require further investigation.

## 5 Conclusion

This study showed that *A. smithii* patches formed fertile islands and led to soil microorganism heterogeneity. The *A. smithii* patches increased the diversity of bacterial communities within the patch but not of fungal communities. The diversity of the fungal community increased in the 0–1 m soil. More Actinobacteria were concentrated in and around the patches compared to non-patch grasslands. Bacterial community diversity was driven by pH, urease, NO_3_^−^-N, and MBC. The contents of SWC, SOM, urease, NO_3_^−^-N, and MBC were the main factors influencing the diversity of the fungal community. This study clarified the effects of *A. smithii* patch formation on grassland vegetation and soil microbial heterogeneity, but further research is still needed on the causes of *A. smithii* patch formation and expansion, such as allelopathy and resource competition.

## Data availability statement

The datasets presented in this study can be found in onlinerepositories. The names of the repository/repositories and accessionnumber(s) can be found below: https://www.ncbi.nlm.nih.gov/, accession number PRJNA1095862.

## Author contributions

HY: Writing – review & editing, Writing – original draft, Software, Methodology, Investigation, Formal Analysis, Data curation, Conceptualization. XY: Writing – review & editing, Writing – original draft, Supervision, Investigation, Funding acquisition. JS: Writing – review & editing, Software, Methodology, Investigation, Formal Analysis. JW: Writing – review & editing, Writing – original draft, Methodology.
