# Supplementary material for: Artemisia smithii patches form fertile islands and lead to heterogeneity of soil bacteria and fungi within and around the patches in alpine meadows of the Qinghai-Tibetan Plateau
Source: Front Plant Sci. 2024 Jun 28;15:1411839. doi: 10.3389/fpls.2024.1411839 (PMC11239433; doi:10.3389/fpls.2024.1411839)
Supplement: Supplementary file 1 [file DataSheet_1.docx]

***Artemisia smithii* patches form fertile islands and lead to heterogeneity of soil bacteria and fungi within and around the patches in alpine meadows of the Qinghai-Tibetan Plateau**

Hang Yang^a, b^, Xiaojun Yu ^a, b, *^, Jianchao Song^a, b^, Jianshuang Wu^b, c^

^a^ College of Grassland Science, Gansu Agricultural University, Lanzhou 730070, China;

^b^ Key Laboratory of Grassland Ecosystem, Ministry of Education, Gansu Agricultural University, Lanzhou 730070, China;

^c^ Institute of Environment and Sustainable Development in Agriculture, Chinese Academy of Agricultural Sciences, Beijing 100081, China.

^*^ Corresponding Author:

Xiaojun Yu

No. 1, Yingmen village, Anning district, Lanzhou, 730070, Gansu Province, China

Email address: [yuxj@gsau.edu.cn](mailto:yuxj@gsau.edu.cn)

**Table 1** Descriptive parameters of plant aboveground biomass and α-diversity inside and outside the patches of *Artemisia smithii*. Different letters within the same column indicate significant differences among distances at *p*<0.05.

| Treatment | Aboveground biomass (g m^-2^) | Shannon | Simpson | Richness | Evenness |
| --- | --- | --- | --- | --- | --- |
| 0 m | 343.82±58.45a | 2.62±0.02b | 0.86±0.01b | 25.33±0.58a | 0.81±0.01b |
| 0-1 m | 125.78±29.66b | 3.02±0.13a | 0.93±0.01a | 24.33±3.06a | 0.95±0.02a |
| 1-2 m | 122.57±30.75b | 3.04±0.08a | 0.92±0.01a | 23.33±1.15a | 0.96±0.03a |
| 2-3 m | 134.54±24.20b | 2.98±0.04a | 0.93±0.01a | 24.00±0.00a | 0.94±0.01a |
| >30 m | 149.32±27.02b | 2.97±0.02a | 0.93±0.00a | 22.33±1.15a | 0.96±0.01a |

**Table 2** Descriptive parameters of plant species importance value inside and outside the patches of *A. smithii*.

| Plant species | | Plant species important values | | | | |
| --- | --- | --- | --- | --- | --- | --- |
|  |  | 0 m | 0-1 m | 1-2 m | 2-3 m | >30 m |
| Gramineae | *Elymus nutans* | 0.073 | 0.071 | 0.052 | 0.066 | 0.086 |
|  | *Poa alpigena* | 0.038 | 0.053 | 0.032 | 0.036 | 0.053 |
|  | *Stipa aliena* | 0.079 | 0.055 | 0.088 | 0.060 | 0.104 |
|  | *Koeleria litvinowii* | *0.016* | *-* | *-* | *-* | *0.002* |
|  |  |  |  |  |  |  |
| Cyperaceae | *Carex capillifolia* | 0.034 | 0.029 | 0.036 | 0.038 | 0.090 |
|  | *Carex alatauensis* | 0.050 | 0.078 | 0.084 | 0.107 | 0.104 |
|  |  |  |  |  |  |  |
| Compositae | *Artemisia smithii* | 0.318 | 0.027 | 0.021 | 0.034 | - |
|  | *Leontopodium nanum* | 0.010 | 0.030 | 0.059 | 0.039 | 0.021 |
|  | *Taraxacum mongolicum* | 0.006 | 0.012 | 0.003 | 0.001 | 0.014 |
|  | *Saussurea katochaete* | 0.002 | - | - | - | 0.017 |
|  | *Anaphalis lactea* | 0.016 | 0.021 | 0.026 | 0.090 | 0.037 |
|  | *Ajania tenuifolia* | 0.016 | 0.027 | 0.020 | 0.017 | 0.020 |
|  |  |  |  |  |  |  |
| Others | *Gentiana lawrencei var. farreri* | - | 0.018 | - | 0.009 | - |
|  | *Anemone obtusiloba* | 0.010 | 0.039 | 0.098 | 0.084 | 0.050 |
|  | *Gentiana macrophylla* | 0.056 | 0.104 | 0.127 | 0.090 | 0.078 |
|  | *Gentiana pseudoaquatica* | 0.003 | 0.007 | 0.007 | 0.009 | 0.037 |
|  | *Potentilla saundersiana* | 0.011 | 0.010 | 0.018 | 0.019 | 0.016 |
|  | *Argentina anserina* | - | 0.007 | - | - | - |
|  | *Galium spurium* | 0.011 | 0.015 | 0.022 | 0.039 | 0.028 |
|  | *Lysimachia maritima* | 0.010 | 0.012 | 0.018 | 0.022 | 0.026 |
|  | *Oxytropis.* sp | 0.027 | 0.063 | 0.055 | 0.038 | 0.071 |
|  | *Astragalus polycladus* | 0.010 | 0.021 | 0.019 | 0.023 | 0.046 |
|  | *Carum buriaticum* | 0.016 | 0.029 | 0.043 | 0.021 | 0.012 |
|  | *Lancea tibetica* | 0.015 | 0.024 | 0.028 | 0.036 | 0.033 |
|  | *Cynodon dactylon* | 0.015 | - | - | - | - |
|  | *Dasiphora fruticosa* | - | 0.067 | 0.066 | 0.043 | 0.012 |
|  | *Plantago asiatica* | - | - | - | 0.009 | - |
|  |  |  |  |  |  |  |
| Poisonous and | *Delphinium grandiflorum* | 0.018 | - | - | - | - |
| harmful plants | *Pedicularis kansuensis* | 0.019 | 0.031 | 0.029 | 0.034 | 0.021 |
|  | *Stellera chamaejasme* | 0.026 | 0.037 | 0.048 | 0.036 | 0.042 |
|  | *Morina kokonorica* | 0.037 | 0.078 | 0.041 | 0.004 | 0.010 |
|  | *Ligularia virgaurea* | 0.059 | 0.129 | 0.098 | 0.088 | 0.052 |
|  | *Aconitum pendulum* | 0.086 | 0.022 | 0.007 | - | - |
|  | *Gymnaconitum gymnandrum* | 0.015 | 0.021 | - | - | - |

**Table 3** Descriptive parameters of soil variables inside and outside the patches of *Artemisia smithii.*

| Treatment | pH | SWC | TN | TP | SOM | AN | AP | NO₃⁻-N | NH₄⁺-N | Urease | Phosphatase | MBC | MBN | MBP |
| --- | --- | --- | --- | --- | --- | --- | --- | --- | --- | --- | --- | --- | --- | --- |
|  |  | (%) | g kg^-1^ | g kg^-1^ | g kg^-1^ | mg kg^-1^ | mg kg^-1^ | mg kg^-1^ | mg kg^-1^ | mg g^-1^ h^-1^ | mg g^-1^ h^-1^ | mg kg^-1^ | mg kg^-1^ | mg kg^-1^ |
| 0 m | 7.14±  0.02a | 17.53±0.28a | 4.37±  0.20a | 1.34±  0.18a | 81.09±2.82a | 296.63±3.23a | 8.10±  1.36ab | 19.44±  0.93a | 2.80±  0.34a | 0.15±  0.01a | 0.04±  0.00a | 434.37±70.35ab | 228.88±21.25a | 11.38±  1.63a |
| 0-1 m | 6.96±  0.01b | 16.38±0.35b | 4.20±  0.28a | 1.51±  0.19a | 74.91±1.16ab | 262.06±4.55bc | 8.86±  0.62ab | 16.60±  0.32b | 2.44±  0.38ab | 0.08±  0.01b | 0.04±  0.00a | 312.02±42.61ab | 165.33±10.62b | 9.94±  1.04a |
| 1-2 m | 6.98±  0.02b | 15.44±0.14bc | 4.41±  0.25a | 1.39±  0.26a | 74.97±1.70ab | 275.77±15.97ab | 10.93±  0.80a | 16.17±  0.15bc | 2.12±  0.33ab | 0.08±  0.01b | 0.04±  0.00a | 274.89±23.87b | 163.53±6.19b | 11.34±  0.88a |
| 2-3 m | 7.13±  0.02a | 15.05±0.28c | 3.74±  0.09ab | 1.34±  0.19a | 70.61±1.06b | 240.62±3.53c | 9.67±  0.48ab | 14.63±  0.16c | 1.22±  0.29b | 0.07±  0.01b | 0.03±  0.00a | 351.43±11.09ab | 180.58±13.98ab | 4.13±  1.43b |
| >30 m | 7.00±  0.03b | 16.31±0.10b | 3.23±  0.11b | 0.98±  0.08a | 70.97±0.79b | 249.5±  4.18bc | 7.13±  0.49b | 19.10±  0.06a | 1.90±  0.25ab | 0.07±  0.00b | 0.04±  0.00a | 473.38±38.99a | 184.95±8.39ab | 9.69±  1.25a |
| ANOVA |  |  |  |  |  |  |  |  |  |  |  |  |  |  |
| *F* | 16.53 | 15.00 | 6.29 | 1.13 | 6.41 | 7.745 | 3.17 | 20.37 | 3.51 | 35.42 | 1.24 | 3.84 | 4.02 | 5.51 |
| *p* | **<0.001** | **<0.001** | **0.001** | 0.364 | **0.001** | **<0.001** | **0.031** | **<0.001** | **0.021** | **<0.001** | 0.319 | **0.014** | **0.012** | **0.003** |

Data are means ± standard errors. Different letters within the same column indicate significant differences among distances at *p*<0.05. SWC, soil water content; TN, total nitrogen; TP, total phosphorus; SOM, soil organic matter; AN, alkali-hydrolyzable nitrogen; AP, available phosphorus; NO₃⁻-N, nitrate nitrogen; NH₄⁺-N, Ammonia nitrogen; MBC, microbial biomass carbon; MBN, microbial biomass nitrogen; MBP, microbial biomass phosphorus.

**Table 4** Topological features descriptive of soil bacterial and fungal community inside and outside the patches of *Artemisia smithii.*

|  |  |  | Bacteria |  |  |  |  |  | Fungi |  |  |
| --- | --- | --- | --- | --- | --- | --- | --- | --- | --- | --- | --- |
|  | 0 m | 0-1 m | 1-2 m | 2-3 m | >30 m |  | 0 m | 0-1 m | 1-2 m | 2-3 m | >30 m |
| Nodes | 98 | 100 | 99 | 100 | 100 |  | 99 | 99 | 100 | 99 | 99 |
| Edges | 303 | 310 | 307 | 289 | 397 |  | 237 | 254 | 238 | 244 | 236 |
| Positive (%) | 50.17 | 51.29 | 53.42 | 55.02 | 54.92 |  | 70.42 | 69.69 | 72.69 | 75.82 | 69.92 |
| Negative (%) | 49.83 | 48.71 | 46.58 | 44.98 | 45.09 |  | 29.54 | 30.31 | 27.31 | 24.18 | 30.08 |
| Average  Connectivity Degree | 3.09 | 6.2 | 6.2 | 5.78 | 7.94 |  | 4.78 | 5.13 | 4.76 | 4.93 | 4.77 |


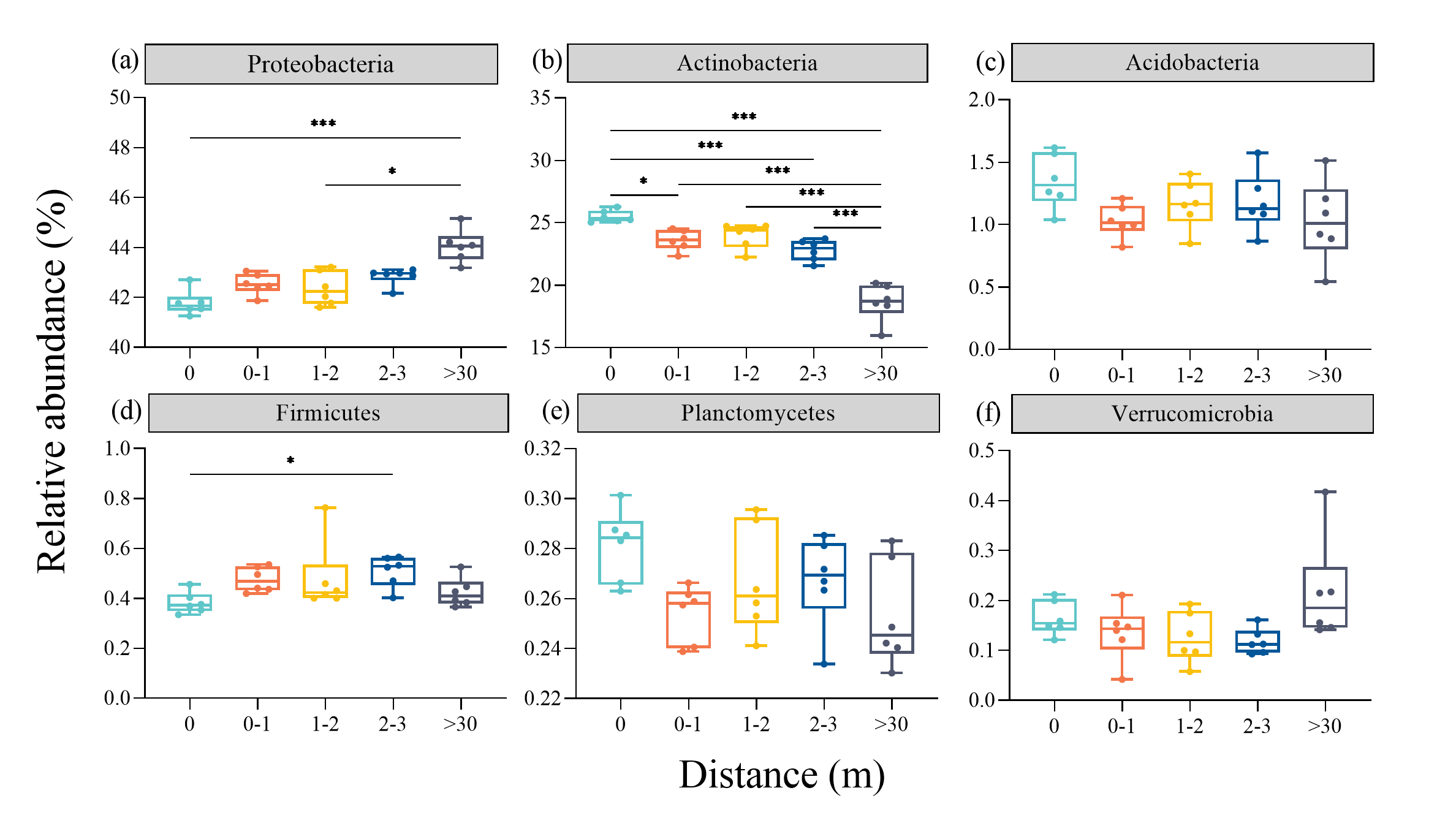


**FIGURE S1** The relative abundance of the dominant bacterial community at the phylum inside and outside the patches of *Artemisia smithii* (*, *p*<0.05; **, *p*<0.01; ***, *p*<0.001)*.*


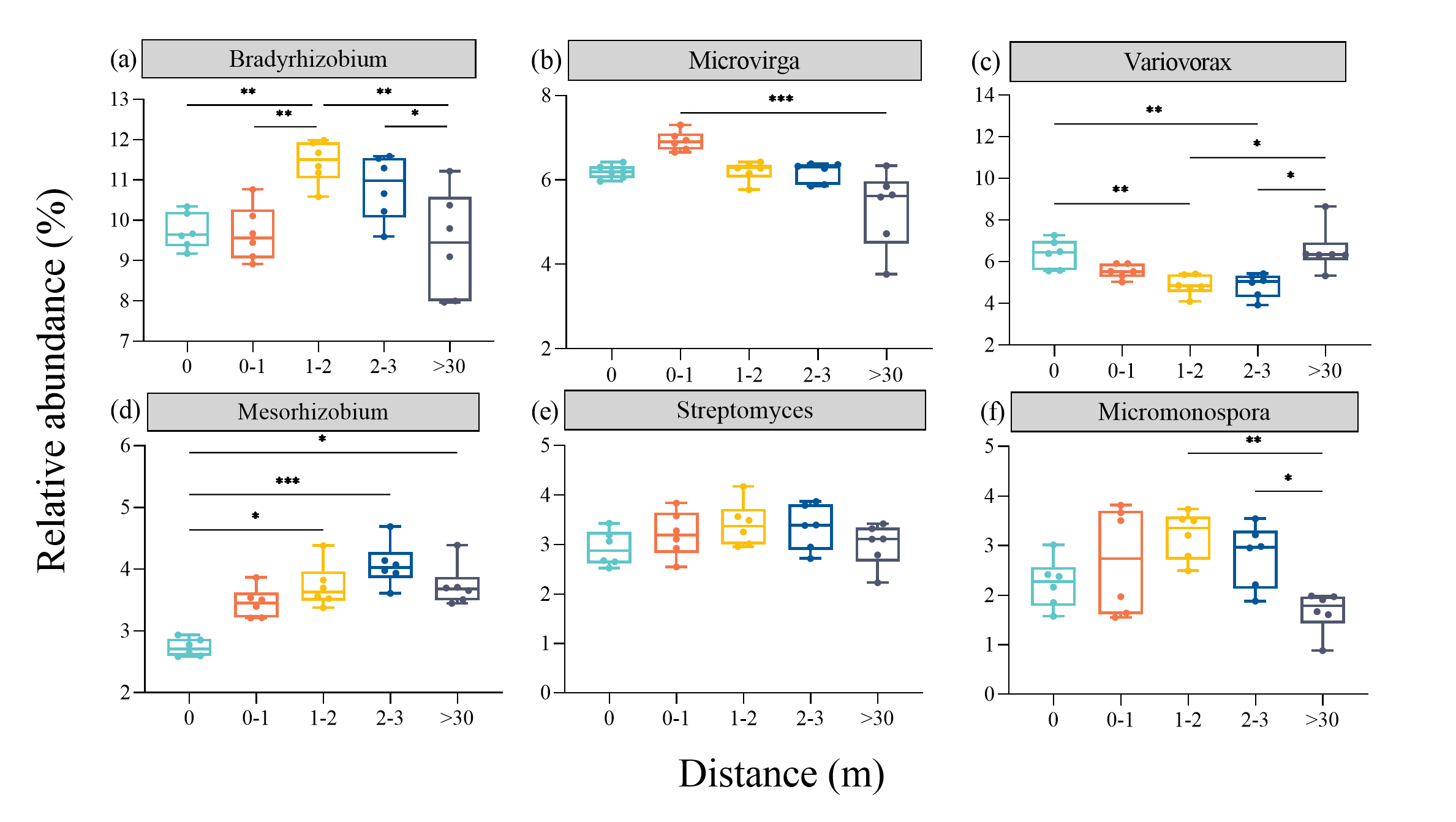


**FIGURE S2** The relative abundance of the dominant bacterial community at the genus inside and outside the patches of *Artemisia smithii* (*, *p*<0.05; **, *p*<0.01; ***, *p*<0.001)*.*


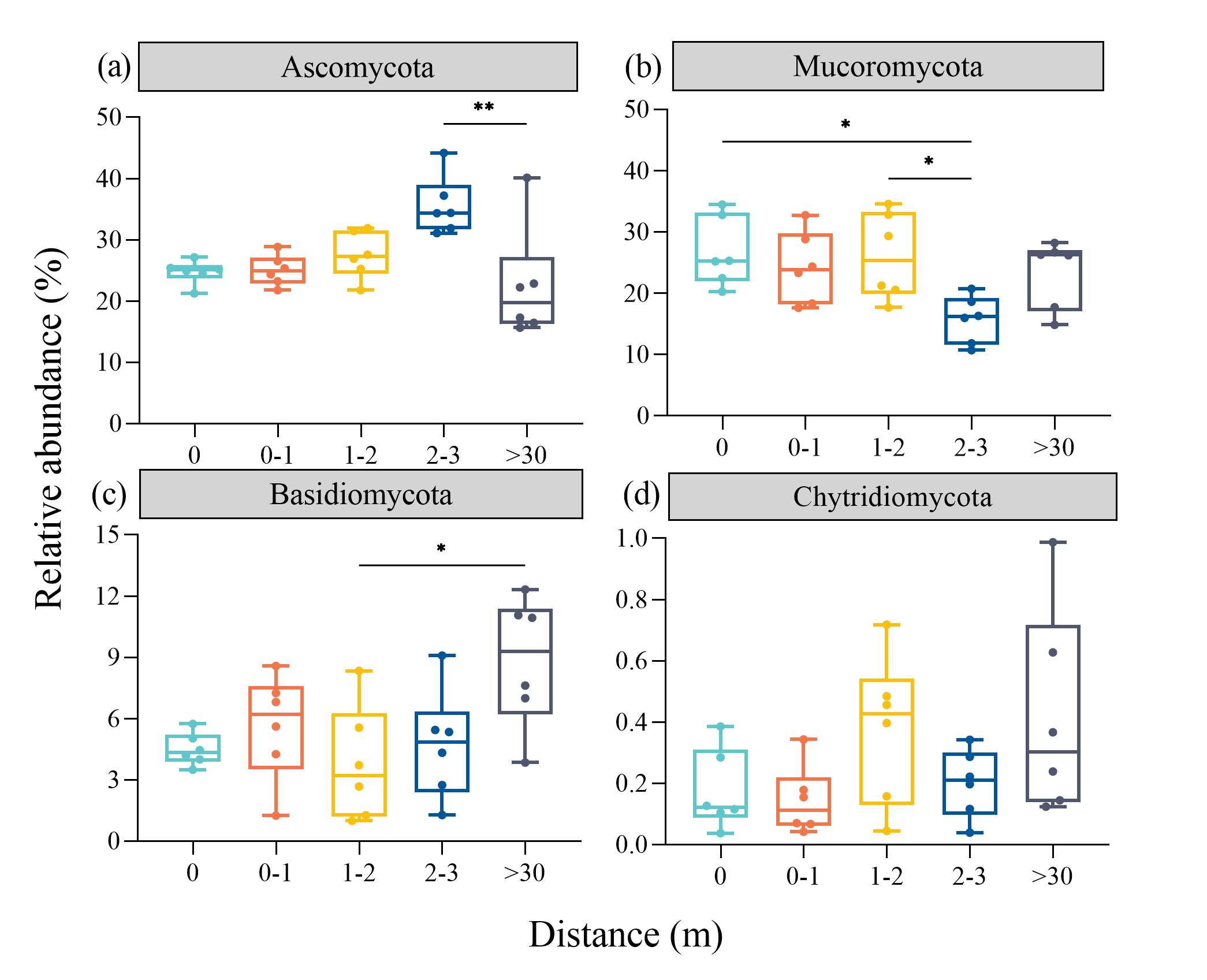


**FIGURE S3** The relative abundance of the dominant fungal community at the phylum inside and outside the patches of *Artemisia smithii* (*, *p*<0.05; **, *p*<0.01; ***, *p*<0.001)*.*


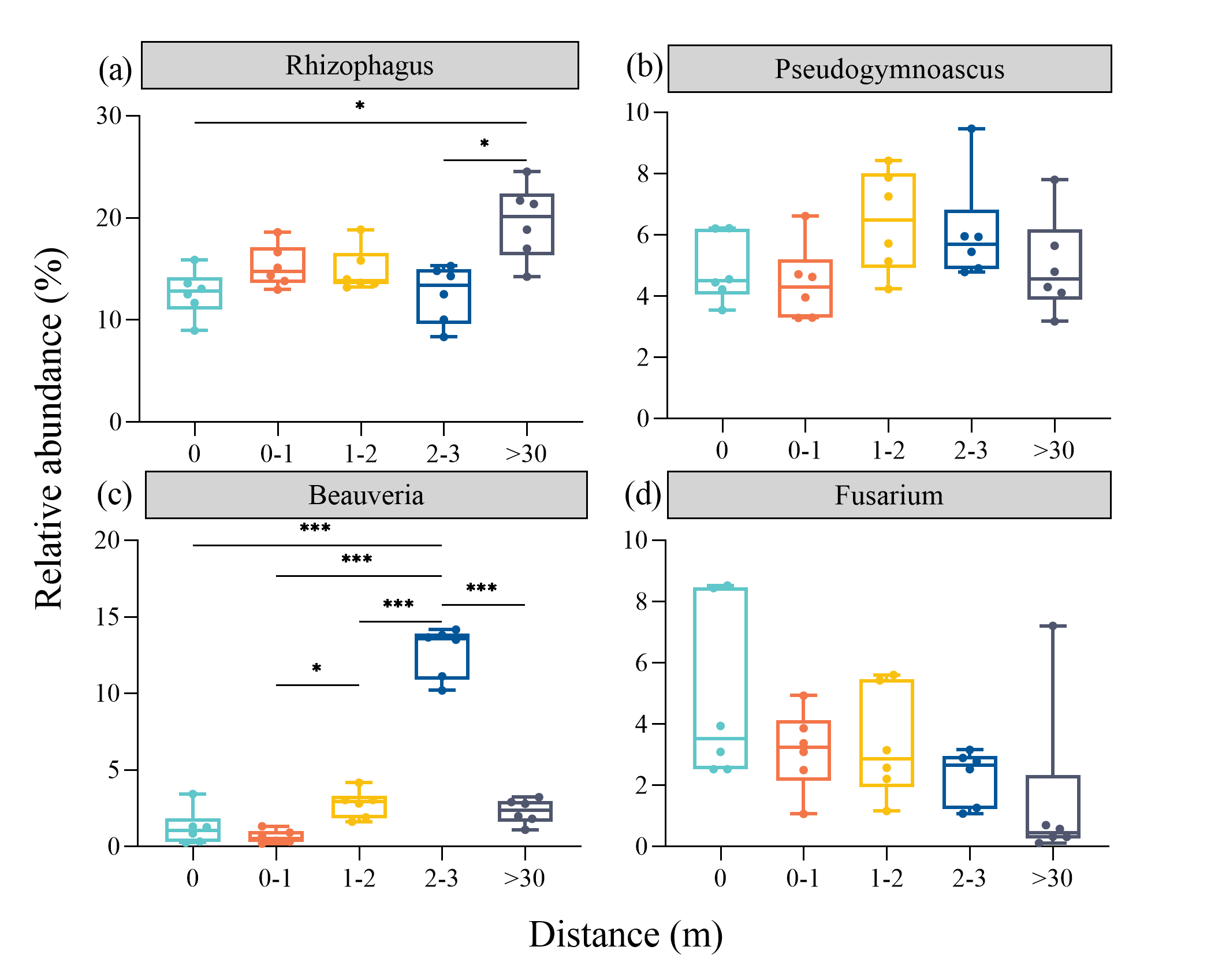


**FIGURE S4** The relative abundance of the dominant fungal community at the genus inside and outside the patches of *Artemisia smithii* (*, *p*<0.05; **, *p*<0.01; ***, *p*<0.001)*.*





**FIGURE S5** The redundancy analysis (RDA) of the bacterial (a, b) and fungal (c, d) communities at the phylum and genus level with environmental factors inside and outside the patches of *Artemisia smithii.* SWC, soil water content; TN, total nitrogen; TP, total phosphorus; SOM, soil organic matter; AN, alkali-hydrolyzable nitrogen; AP, available phosphorus; NO₃⁻-N, nitrate nitrogen; NH₄⁺-N, Ammonia nitrogen; MBC, microbial biomass carbon; MBN, microbial biomass nitrogen; MBP, microbial biomass phosphorus.
